# Glycosaminoglycans: Carriers and Targets for Tailored Anti-Cancer Therapy

**DOI:** 10.3390/biom11030395

**Published:** 2021-03-08

**Authors:** Aikaterini Berdiaki, Monica Neagu, Eirini-Maria Giatagana, Andrey Kuskov, Aristidis M. Tsatsakis, George N. Tzanakakis, Dragana Nikitovic

**Affiliations:** 1 Laboratory of Histology-Embryology, School of Medicine, University of Crete, 71003 Heraklion, Greece; berdiaki@uoc.gr (A.B.); eirini_gt@hotmail.com (E.-M.G.); tzanakak@uoc.gr (G.N.T.); 2 Department of Immunology, Victor Babes National Institute of Pathology, 050096 Bucharest, Romania; neagu.monica@gmail.com; 3 Department of Technology of Chemical Pharmaceutical and Cosmetic Substances, D. Mendeleev University of Chemical Technology of Russia, 125047 Moscow, Russia; a_n_kuskov@mail.ru; 4 Laboratory of Toxicology, School of Medicine, University of Crete, 71003 Heraklion, Greece; tsatsaka@uoc.gr; 5 Laboratory of Anatomy, School of Medicine, University of Crete, 71003 Heraklion, Greece

**Keywords:** glycosaminoglycans, cancer, cancer therapy, hyaluronan, heparan sulfate, heparin, chondroitin sulfate, drug carriers, nanomaterial, therapy targets

## Abstract

The tumor microenvironment (TME) is composed of cancerous, non-cancerous, stromal, and immune cells that are surrounded by the components of the extracellular matrix (ECM). Glycosaminoglycans (GAGs), natural biomacromolecules, essential ECM, and cell membrane components are extensively altered in cancer tissues. During disease progression, the GAG fine structure changes in a manner associated with disease evolution. Thus, changes in the GAG sulfation pattern are immediately correlated to malignant transformation. Their molecular weight, distribution, composition, and fine modifications, including sulfation, exhibit distinct alterations during cancer development. GAGs and GAG-based molecules, due to their unique properties, are suggested as promising effectors for anticancer therapy. Considering their participation in tumorigenesis, their utilization in drug development has been the focus of both industry and academic research efforts. These efforts have been developing in two main directions; (i) utilizing GAGs as targets of therapeutic strategies and (ii) employing GAGs specificity and excellent physicochemical properties for targeted delivery of cancer therapeutics. This review will comprehensively discuss recent developments and the broad potential of GAG utilization for cancer therapy.

## 1. Introduction

The tumor microenvironment (TME) is composed of cancerous, non-cancerous, stromal, and immune cells that are surrounded by the components of the extracellular matrix (ECM) [1]. The ECM is a significant component of the TME with a vital role in cancer’s pathogenesis [2,3]. It is well established that TME plays an essential role in tumorigenesis. Indeed, tumor growth and metastasis steps, e.g., primary lesion development, intravasation, extravasation, and metastasis to anatomically distant sites, are executed via the discrete interplay of the tumor cells with their microenvironment [4]. Glycosaminoglycans (GAGs), natural biomacromolecules, and essential ECM and cell membrane components are extensively altered in cancer tissues [5]. Indeed, these heteropolysaccharides vital in supporting homeostasis have also been established to participate in inflammatory, fibrotic, and pro-tumorigenic processes [6,7,8,9]. Both free GAGs and GAGs bound into the protein cores of proteoglycans- (PG) are crucial mediators of cellular and ECM microenvironments, with the ability to specifically bind and regulate the function of ligands and receptors crucial to cancer genesis [4,10,11].

Structurally, GAGs are linear, long-chained polysaccharides consisting of repeating disaccharide units linked by glycosidic bonds. These building blocks are composed of N-acetylated hexosamine and uronic acid. The type of the disaccharide repeating unit and its modifications, including discrete sulfation patterns, allows the classification of GAGs into specific categories, e.g., chondroitin sulfate (CS)/dermatan sulfate (DS), heparin (Hep)/heparan sulfate (HS), keratan sulfate (KS) and hyaluronan (HA) [12,13,14,15]. KS chains contain galactose instead of uronic acid in their disaccharide building blocks [15]. CS/DS, HS/Hep, and KS chains are covalently bound into the protein cores of proteoglycans [6]. On the other hand, the non-sulfated GAG HA is not bound into the proteoglycan core but is secreted to the ECM of almost all tissues [13].

Bound GAGs are initially synthesized on core proteins at the Golgi lumen. Their glucuronic acid—N-acetylglucosamine/N-acetylgalactosamine(GlcA-GlcNAc/GalNAc) or, in the case of KS, galactose-N-acetylglucosamine (Gal-GlcNAc) repeating units are subjected to significant structural modification, including sulfation and in the case of HS/CS epimerization at the Golgi apparatus. Moreover, the desulfationof HS chains is performed at the cell membrane compartment [16]. The fine modifications result in an astonishing number of divergent GAG structures.

The GAG fine modifications define, to no small degree, the specificity of their binding with proteins. Notably, GAGs have been shown to interact with more than 500 proteins [17]. The interactions of GAGs with membrane receptors, ECM proteins, chemokines, and cytokines, as well as enzymes and enzyme inhibitors, are crucial in both development and homeostasis [18,19]. Likewise, GAGs’ interactions with the above, both soluble and insoluble ligands, play a vital role in various diseases, including cancer [20]. By modulating numerous signaling pathways, GAGs exert distinct effects on cancer cells’ functions, cancer stroma interactions, and cancer-associated inflammation, thus regulating essential processes for tumor progression and metastasis [1,4,6,21]. 

During disease progression, the GAG fine structure changes in a manner associated with disease evolution. Thus, changes in the GAG sulfation pattern are immediately correlated to malignant transformation [22]. Their molecular weight, distribution, composition, and subtle modifications, including sulfation, exhibit distinct alterations during cancer development [23,24]. Thus, most tumor types exhibit increased CS content with an increase in the 6-O-sulfated and/or unsulfated disaccharide content and a decrease in the 4-O-sulfation level due to changes in relevant enzyme activities [23,24]. Likewise, an aberrant HS sulfation pattern has been correlated to tumorigenesis. It was shown that the *N*-sulfation of GlcNresidues in specific domains along the HS chain facilitate tumor angioegenesis [25]. The expression of HS 6*O*-sulphated disaccharide content was shown to be increased during chondrosarcoma [26] and colon carcinoma progression [27].

GAGs and GAG-based molecules, due to their unique properties, are suggested as promising effectors for anticancer therapy [28]. Considering their participation in tumorigenesis, their utilization in drug development has been the focus of both industry and academic research efforts [29]. These efforts have been developing in two main directions; (i) utilizing GAGs as targets of therapeutic strategies and (ii) employing GAGs exquisite specificity and excellent physicochemical properties for targeted delivery of cancer therapeutics.

This review will discuss recent developments and the broad potential of GAG utilization for cancer therapy.

## 2. Focus on GAGs’ Structure and Roles

GAG polymers are assembled through several consecutive steps with different enzymes’ involvement at each separate stage. Sulfated GAGs are synthesized by specific enzymes in the cell’s Golgi apparatus, whereas HA is synthesized by transmembrane proteins called HA synthases (HASs). While HA is not linked to a protein and is produced from its reducing end, the sulfated GAGs are built up from the non-reducing end and synthesized as side chains attached to a protein core of PGs [5].

In the case of KS, GlcA is replaced by GalN. Henceforth, the growing GAG chain’s modifications, e.g., deacetylation/N-sulfation and epimerization of GlcA to IdoA followed by O-sulfation, are performed [30,31]. Therefore, the individualized functionalization of GAGs results in their unique structures. Indeed, distinct sulfation patterns have been identified at the disaccharide unit’s functionalization sites, hexosamine, and IdoA components, facilitating great complexity and structural diversity [32,33].

Different variations in the expressions/activities of enzymes involved in GAG synthesis have been described. One example is that the levels of exostoses (multiple)-like 1 (EXTL1) and CS N-acetylgalactosaminyltransferase 1 (CSGalNAcT-1), which participate in the production of HS and CS, respectively, were shown to exhibit an inverse ratio of expression. The inverse expressions identified in the process of B-cell differentiation have been suggested to act as a switch enabling either CS or HS synthesis observed during these cell differentiations [34].

### 2.1. Heparin and Heparan Sulfate

Both Hep and HS chains are synthesized as a modification of a PG protein core, sharing a biosynthetic scheme but exhibiting some disparities [35,36]. Thus, initially, the sequential addition of four sugar residues by different glycosyltransferases will give rise to the linker tetrasaccharide (for Hep/HSXyl-Gal-Gal-GlcA) connected to the core protein’s serine residue as a linker region [37]. Notably, the linkage region also serves as a primer for the initiation of the CS chains biosynthesis. In the case of HS, the members of the EXTL family of glycosyltransferases trigger chain creation by transferring an *N*-acetylglucosamine (GlcNAc), whereas in the case of CS chains, a β-*N*-acetylgalactosamine (β-GalNAc) residue is attached to the linkage primer by a CSGalNAc-transferase [37]. Polymerization of HS then takes place by the alternating addition of GlcAβ1,4 and GlcNAcα1,4 residues through the action of designated glycosyltransferases [38].Modifications, such as N-deacetylation and N-sulfation of glucosamine, and O-sulfations are subsequently performed. The GlcA residues can, on some occasions, be epimerized to iduronic acid (IdoA)[35,36].

The two GAGs differ, as the main HS disaccharide unit comprises a GlcA and N-acetylated GlcN(GlcNAc). In contrast, the main Hep disaccharide consists of sulfated, at the carbon 2 IdoA(IdoA2S), and N-sulfated GlcN also sulfated at C6 (GlcNS6S). Due to the high Hep sulfation level, this GAG is characterized as a biomacromolecule with the highest negative charge density [39]. The functionalization with sulfate is uniformly distributed along the Hep chain, whereas HS chains exhibit alternatively exchanging regions of high sulfation with lower or non-sulfated sequences [40]. Indeed, Sulf-1 and Sulf-2, sulfatase enzymes, are active at the extracellular compartment and trim the 6-O-sulfates partially from HS, but do not affect Hep, which is not located at the cells’ membranes [41]. As a result, the Hep chain mainly comprises trisulfated disaccharides (80%) consisting of sulfated IdoA and sulfated GlcN.

The HS chains predominantly consist of disaccharide repeats comprised of GlcA and GlcNAc, with a much lower sulfation level [42]. Notably, the “fully sulfated” HS sequences, denominated as S domains, commonly exhibit the highest binding propensity to Hep/HS-binding proteins [43]. Indeed, the binding between proteins and HS/Hep is most commonly executed by charge–charge interactions between the proteins’ basic amino acids and the anionic sulfate and/or carboxylate [18,44]. The interaction between respective binding proteins and HS is likewise affected by the GAG heterogeneity and cationic association [19]. Moreover, posttranslational modifications, such as N-glycosylation, of the HS/Hep binding proteins can regulate ligand and HS/Hep binding as shown for the fibroblast growth factor receptor 1 [45]. Notably, its disaccharide unit’s extensive modifications render HS the most complex animal polysaccharide [19].

HS chains are synthesized by almost all mammalian cells in the forms of HSPG and are localized to the cell membrane (e.g., syndecans) and pericellular space/basement membranes (e.g., perlecan) or extracellular matrices. Despite the HS chain’s extensive functionalization, its fine structure is notably conserved in a given cell type [46,47]. HS’s composition varies both spatially and temporally during development and in a celltype-dependent manner. The involved regulating mechanisms remain poorly elucidated.

Significant changes occur in HS composition during carcinogenesis, and vitally, both tumor growth and tumor-dependent angiogenesis depend on HS growth factor interactions [48].

Hep is synthesized only in connective tissue-type mast cells or basophils [49]. The Hep chain is synthesized during the core protein modification of the PG, seglycin. Seglycine exhibits a small protein core but undergoes extensive glycosylation, resulting in a molecular weight up to 750 kDa [50]. The bound Hep chains’ molecular weight varies between 60 KDa and 75 kDa. These Hep chains are cleaved into 5–25 kDa fragments when mast cells and basophils are degranulated [51,52]. Mast cells release Hep by exocytosis upon binding specific antigens to the IgE antibodies attached to their cell-surface receptors [53]. However, Mast cell serglycin can also be decorated by other GAG chains, such as CS and DS [54].

Hep, however, can be uptaken by various cells, including endothelial cells, as the primary site for removing unfractionated Hep from the circulation is the liver sinus endothelial cells [55].

In mammalians, HS/Hep are enzymatically degraded by heparanase, a strict endo-β-glucuronidase [56].

### 2.2. Chondroitin Sulfate/Dermatan Sulfate

The CS chains consist of disaccharides comprising β(1-4) GlcA and β(1-3) GalNAc. The sulfation pattern of the GlcA and GalNAc determinesthe type of CS. Thus, CS-A is characterized by single sulfation at C4 of the GalNAc, whereas CS-C is determined by single sulfation at C6 of GlcA. Other functionalizations exist, as GalNAc can be sulfated at the carbon 4 and/or 6, whereas GlcA can also be sulfated at the C2 and/or C3 [57,58]. On the other hand, CS-B denominated similarly to DS, consisting of alternating GlcA or IdoA, which can be sulfated at C2, and GalNAc, which can be functionalized by sulfation at C4 or C6 [58]. Both CS and DS exhibit vast differences regarding chain length and MW, with the latter being in the 5–70 kDa range [59]. The prominent heterogeneity of the CS/DS chains is directly correlated to these GAGs’ biological roles [60,61].

An example is the altered functionalization of CS/DS in gastric cancer as the sulfation at C4 is downregulated, and sulfation at C6 increased in tumor cells compared to normal gastric cells. Additionally, the chain length of CS/DS and the GAG content of the PGs, decorin, and versican was decreased significantly.

### 2.3. Keratan Sulfate

The KS chains consist of disaccharides containing β(1-4) GlcNAc and β(1-3) Gal. This specific glycosidic binding results in a GAG chain formation, unique for its lack of a carboxyl group. KS’, binding into the protein core of PGs differs compared to HS/CS. Thus, corneal KS denominated as KS-I binds to an Asn in the core proteins through an N-linked complex, branched oligosaccharide. On the other hand, in cartilage, the KS chains denominated as KS-II utilize their N-Acetylgalactosamine (GalNAc) to establish an O-link with the Ser or Thr residues of the protein cores [62]. The type III KS (KS-III), initially identified in the brain tissue, links a mannose to a Ser residue of the protein core [63]. KS chains have a molecular weight ranging from 5–25 kD [64].

KS structure is mostly dependent on the tissue type as corneal KS-I exhibits longer chain length and a lower degree of sulfation than the cartilage KS-II. KS-III is mainly bound to PGs localized to the brain and nervous tissues [65,66]. The expression of KS is also deregulated in cancer. Indeed, it was suggested that KS’s aberrant expression could be utilized as a marker of pancreatic cancer progression and metastasis [67] and that highly sulfated KS is produced by malignant astrocytic tumors [68].

### 2.4. Hyaluronan

Transmembrane enzymes denominated HA synthases (HAS) produce HA chains. The three HAS isoforms, HAS1, HAS2, and HAS3, use cytoplasmic UDP-glucuronic acid and UDP-N-acetylglucosamine as substrates. Their active site is localized intracellularly, whereas the synthesized HA chain extrudes into the ECM [13]. This non-sulfated GAG is composed of repeating units of GlcNAc and GlcA combined by β-1.3 and β-1.4 linkages, with an average mass of 100–2000 kDa [13]. HAS1 and HAS2 synthesize a high molecular weight polymer, whereas HAS3 produces shorter chains (~2 × 10^6^ Da vs. ~2 × 10^5^ Da, respectively) [69]. HA’s biological information is translated to the length of its polymers and defines its effects [70]. The UDPsugar precursors and holistic cell metabolism responsible for producing HAS substrates critically regulate HASs activities [71]. HA-mediated effects are executed through various mechanisms that involve the binding of HA to surface receptors such as CD44 and RHAMM [72,73,74] and the internalization of HA through receptor-mediated endosomal pathways [75].

The human genome contains five active hyaluronidases (Hyals) (Hyal1–Hyal4 and PH-20) and the non-transcribed Hyal pseudogene (HyalP1). Hyal 2 and 3 exhibit degrading activity, exclusively for HA [76]. Some human Hyals exhibit degrees of CS-degrading activity. Thus, PH20 shows high activity for HA and low CS-degrading activity. On the other hand, Hyal1 degrades CS-A more swiftly than HA [77]. Hyal-4 is misnamed, as it shows specificity for CS and no ability to degrade HA [78].

Hyal1 is widely expressed and localized to lysosomes or trafficking vesicles [79]. However, Hyal 1 can also be secreted to the ECM by tumor cells [80]. Hyal1 is upregulated in many human cancers and has been correlated with tumorigenesis [81].

In contrast, Hyal2 is bound onto the cell membrane via a GPI anchor and is usually associated with lipid rafts [82], wherein, in common with CD44 and Hyal1, it promotes HA cellular uptake and endocytic internalization [75].

## 3. Types of Nanoparticles and Materials Utilized for Targeted Drug Delivery—Focus on GAG-Based Nanoparticles

The development of targeted delivery systems for anticancer drugs in the form of nanoparticles has been prioritized since classical methods, namely chemotherapy, radiation therapy, surgery, and their combination, still do not benefit a significant number of patients [83].

Micro- and nanoencapsulation [84,85,86], micellar [87,88], and liposomal [89] forms, dendrimers [90], mesoporous particles [91], and nanogels [92] are used most often for targeted drug delivery. A wide range of compounds, both synthetic [93,94] and natural [86,87,89,92], are used as materials, and each of the groups has several advantages and disadvantages (Table 1).

The delivery of nanoparticles to the tumors rests on a series of both specific and nonspecific interactions with cells. The specific interactions are based on functionalizing the surface of nanoparticles with ligands that are specific for the target tumor tissue, including tumor cells, intracellular targets, intratumoral and peritumoral blood vessels, and the ECM. The nonspecific nanoparticles are coated solely with stabilizing agents. Most of the studies suggest that the crossing of the tumor blood vessel barrier by nanoparticles is mostly perpetrated through intercellular gaps. Their restraint to the tumor site is dependent on the pressure produced by inadequate lymphatic drainage, commonly denominated as the enhanced permeability and retention (EPR) process [95]. Recent developments suggest that more than 90% of nanoparticles actively enter solid tumor tissue through endothelial cells, challenging the current rationale for nanomaterial synthesis [96]. Nanoparticles targeting specific tumor-associated antigens exhibit superior delivery and effects [96]. A new stage in developing nanomaterials is utilizing patient-derived macromolecules, as recently shown by Lazarovits et al. [97].

In common with others, GAG-based nanoparticles have to overcome the mononuclear phagocytic system’s action, which attenuates their efficiency through sequestration and elimination. Notably, nanoparticles carrying a negative charge are more prone to phagocytosis than positive surface charge carrying nanoparticles. Thus, modulating CS charges with competent functionalization can attenuate their phagocytosis [98]. Renal excretion function is another obstacle as it can severely attenuate nanoparticles’ actual delivery efficiency. Indeed, renal excretion function seems to be facilitated by incorporating GAG components even though it does not seem to affect tumor accumulation [99]. Modification of the hydrodynamic diameter to the 5.5 nm–100 nm range minimizes kidney excretion and enhances delivery efficiency [100].

Nanocarriers obtained using biocompatible natural polymers such as GAGs do not exhibit adverse effects on cell viability in cell cultures. They show good biocompatibility in animal experiments [92,101,102]. In addition to biocompatibility and specificity, GAG-based nanocarriers, when their GAG components are specifically modified, exhibit other properties, such as high stability, adjustable particle size, and the ability to respond to external stimuli, such as temperature, light, pH, and ionic strength [103,104,105], enabling multifunctional utilization [106,107]. GAGs, such as CS and HA, have been utilized as therapeutic agents for various pathologies, including osteoarthritis [108,109], with no significant side effects, suggesting their long-term safety. The broad utilization of HA in dermatological clinical practice has not been associated with side effects [110].

The resulting nano-systems’ properties depend on the type and concentration of polymer used for their production and the type and degree of intermolecular interaction or crosslinking. Thus, HA can generate self-assembling micelles with the ability to create amphiphilic nanocarriers. Indeed, HA micelles can effectively deliver hydrophobic drugs to target cancer cells while simultaneously facilitating bioavailability and the half-life of the utilized drugs [111]. Importantly, nanoparticles can be loaded with various types of drugs, both hydrophilic and lipophilic, as well as DNA, RNA, peptides, and proteins [85,88,112,113].

**Table 1:** Types of nanoparticles and materials utilized for targeted drug delivery.

Nanoparticle System	Material	Nanocarriers Type	Examples of Carried Agents	Reference
Lipids	Phospholipids	Liposomes, solid lipid particles	RGD peptide, apatinib	[89]
Synthetic polymers	Poly(N-isopropylacrylamide, poly-N-vinylpyrrolidone, poly(lactic-co-glycolic acid)	Micelles, nanoparticles,	Doxorubicin, curcumin, indocyanine green	[85,88,93,94,104,112]
Natural polymers	HA, alginate, chitosan, heparosan, carboxymethyl starch, CS, Hep	Microcapsules nanospheres, nanoparticles, nanogel, micelles	Doxorubicin, BSA, tirapazamine, cisplatin	[84,85,86,87,92,101,106,111] Section 1
Dendrimer	Polyester, Polyacetal/polyketal	Micelles	Camptothecin, methotrexate	[90]
Silica	Mesoporous silica	Nanoparticles	Doxorubicin, fluorescein isothiocyanate	[91]
Metal	Gold	Nanoparticles, nanorods	Doxorubicin, bleomycin	[105]

### 3.1. Heparin and Heparan Sulfate for Anticancer Drug Delivery

Hep and low-molecular-weight heparins (LMWHs) are widely used as a clinical anticoagulant due to their ability to bind with and inhibit the serine-threonine antithrombin protease [114]. Hep is also studied and used for applications in other therapeutic areas due to its biocompatibility, for example, wound healing, burn injury treatment, inhibition of inflammation, and metastatic spread of tumor cells [115]. Hep’s chemical and physical properties connected with the large surface area of its chain and the presence of reactive functional groups allow efficient binding of different anti-tumor agents. Nanoparticles based on Hep can be applied as efficient anticancer agent carriers with versatile surface chemistry for functionalization and the introduction of biomolecules [116]. Some of the Hep derivatives are used to deliver imaging agents, such as iron oxide nanoparticles, to detect tumor cells in humans [117]. Sodium deoxycholate (DOC)-conjugated Hep derivatives (DOC-heparin) were used to prepare nanoparticles for in vivo tumor targeting and inhibition of angiogenesis based on chemical conjugation and the EPR effect [118]. More substantial anti-tumor effects of the DOC-heparin were achieved in animal studies compared to Hep alone. Obtained results confirmed that the conjugated Hep retained its ability to inhibit binding with the angiogenic factors, inducing a significant decrease in endothelial tubular formation. In a separate study, dendronized Hep–doxorubicin (Dox) conjugates were prepared, exhibiting a combination of Dox and Hep features and characterized as pH-sensitive drug delivery vehicles [119]. The prepared nanoparticles showed potent anti-tumor activity, induced apoptosis, and significant antiangiogenesis effects in the 4T1 breast tumor model. Additionally, dendronized Hep and the derived nanoparticles with the loaded drug demonstrated no significant toxicity to the healthy organs of both tumor-bearing and healthy mice, which was confirmed by histological analysis.

Park et al. first attached low molecular weight Hep to stearylamine to obtain amphiphilic polymer that was used to prepare self-assembled micelle-like nanoparticles, loaded with docetaxel in their hydrophobic core. The obtained preparation was tested in MCF-7 and MDAMD 231 human breast cancer cells. This approach demonstrated that Hep retained about 30% of its anticoagulant activity, increased the half-life time of docetaxel in the novel preparation used, and significantly inhibited tested cells’ viability [120]. Park et al. synthesized an amphiphilic biopolymer made of Hep and deoxycholic acid and prepared nanoparticles loaded with Dox. These nanoparticles were tested for cytotoxicity and anti-tumor effects. The investigated system showed high loading efficiency and a substantial anti-tumor effect [121].

Other studies describe the characteristic properties of Hep-based nanoparticles as potential drug delivery systems, not focusing on specific types of cancer [122].

In summary, Hep is capable of forming nanoparticles upon the introduction of amphiphilic or hydrophobic molecules [116,123,124]. It can also interact with proteins, which leads to the formation of complexes with various biological effects [125,126]. Nevertheless, absorption of blood proteins upon the introduction of Hep nanoparticles into the human body needs to be controlled.

#### 3.1.1. Micellar Heparin Nanoparticles

Studies showed that it is necessary to modify the Hep surface of nanoparticles to reduce blood elements’ absorption. Moreover, it is possible to introduce additional specific receptors for targeted delivery directly to the tumor [127,128]. In a study on the development of Hep-based micelles, multifunctional self-assembling nanoparticles were created that combine the following properties: the carrier material is non-toxic, and the resulting micelles had high stability and sensitivity to pH. Intravenous injection of the Hep/Dox combined micelles increased Dox blood circulation time and enhanced its accumulation at the animal model’s tumor site [129].

Hep nanoparticles can penetrate body barriers. Thus, a study showed that Hep particles 100 nm in size effectively overcame the blood brain barrier (BBB), as evidenced by an increase in the concentration of drugs in the brain target tissue [130]. However, particles with a small size very quickly left the circulation, which indicated the need to select the functionalization of their surface specifically.

#### 3.1.2. Heparin-Coated Metal NanoParticles

Another important direction is the development of targeted delivery systems based on magnetic metal nanoparticles. The main disadvantage of such nanomaterials is that we need to select a proper stabilizer or coating that will contribute to the constant particle size, reduce their toxic effects, increase biocompatibility, and overcome physiological barriers maintaining their high magnetic properties. Hep was found to be a sound basis for these coatings [131]. Another study demonstrated that with Hep’s utilization, it is possible to create stable magnetic nanoparticles, based on iron oxide, exhibiting low polydispersity [132]. The introduction of cis-platin to the composition of Hep and iron oxide created Hep-coated metal nanoparticles, which exhibited a cytotoxic effect on cancer cells but lowered toxic side- effects compared to the free drug [133].

In similar studies, magnetic nanoparticles were modified with polyethylene glycol (PEG) and Hep, after which they were functionalized with additional targeting agents. PEGylation enables longer circulation time but can also render metal nanoparticles increased passive targeting via the EPR effect. PEGylated metal nanoparticles were, furthermore, modified with a Hep layer to enable the carrying of the highly cationic CPP-linked protein drug [134]. Further studies demonstrated that the resulting nanoparticles have an increased recirculation time in the blood, retain their high magnetic properties, and overcome the BBB. It was also shown that in a mouse 9L glioma model, particles with a size of more than 50 nm accumulate at high concentrations in the tumor tissues [135].

#### 3.1.3. Heparin Nanogels

Delivery systems based on liposomes, micelles, and magnetic nanoparticles are relatively well-studied systems for which specific rules and dependencies have already been developed, but depot forms based on nanogels represent a new milestone in this field [136]. Most scientific research, in this area, is devoted to creating matrices based on natural polymers, including Hep, chitosan, alginic acid salts, and others.

The majority of the studies were dedicated to delivering genes and proteins. There are also several reports in which Hep nanogels have been developed for the targeted delivery of anticancer drugs [100]. A polymer matrix is typically produced by covalent crosslinking to form strong and stable structures. Due to the polymer’s properties, the delivery system can be sensitive to a wide range of external factors, and thus, fine-tuned release of the drug load can be accomplished [124]. Melanoma is characterized by a high metastatic potential of the transformed melanocytes, which also become “invisible” to the immune cells. This “invisibility” is sustained by many mechanisms, one of them being the formation of a platelet cloak. The heterogeneous mixture of GAGs can inhibit this process by blocking P-selectin-mediated intercellular adhesion. LMWHep-coated with Dox and loaded in liposomes (LMWHep-Dox-Lip) was studied in the B16F10 melanoma cell line. This nanomaterial exerted both a cytotoxic effect and inhibited the adhesion between tumor cells and platelets mediated by P-selectin. It was demonstrated in vivo that the pulmonary metastases of melanoma are prevented by LMWHep-Dox-Lip treatment [137].

This type of drug-delivery system can be utilized for combination chemotherapy, where more than two drugs with different properties and mechanisms of action are applied to boost the cancer treatment. Thus, Joung et al.produced Hep-Pluronic (Hep-Pr) nanogel loaded with paclitaxel and DNAase [138]. The nanogel allowed robust intracellular delivery to facilitate these drugs’ synergistic effects in a dose-dependent manner and inhibited tumor cells’ growth. Notably, the synthesized matrix can bind to high concentrations of both hydrophilic and hydrophobic drugs. Nanogels exhibit some disadvantages due to their high polydispersity, hence the uneven distribution of the active substance in the volume [139].

Some approaches utilize HS for nanoparticle preparation. A recent drug delivery strategy conjugated the chemotherapeutic agent, docetaxel, onto HS. Due to its antimetastatic and T cells infiltration enhancing properties, Aspirin (ASP) was encapsulated into the HS-docetaxel micelle followed by the cationic polyethyleneimine (PEI)-polyethylene glycol (PEG) copolymer binding to HS via electrostatic force. This approach results in an ASP-loaded HS-docetaxel micelle (AHD)/PEI-PEG nanocomplex (PAHD). PAHD exhibits a long half-life in the blood due to the PEG shell. As TME is characterized by weakly acidic pH, the PEI-PEG polymers detach from AHD and increase tumor cells’ permeability due to their positive charge. Heparanase, overexpressed by tumor cells, degrades HS, thus delivering the active ASP and docexatel to tumor cells. Indeed, PAHD exhibits targeted toxicity toward tumor cells but not normal cells and is bestowed with superior ability to suppress tumor growth and lung metastasis in 4T1 breast cancer tumor-bearing mice [140].

#### 3.1.4. Summary

Significant progress in utilizing Hep-based nanoparticles as novel venues or in combination with existing therapies, such as chemotherapy or photodynamic therapy [131,138,141], has been achieved. Indeed, many recent studies have proven that Hep-based nano-scaled systems have great potential as drug carriers, the ability for specific delivery to cancer tissues, and excellent biocompatibility [122].

However, even though significant achievements have been obtained in the synthesis of Hep-based nanoparticles, no such nanomaterials have made their way to clinical trials. A hurdle to clinical transition is the anticoagulant properties ofHep, which can lead to bleeding complications. Chemically modifying Hep can attenuate its anticoagulant activities; however, the mechanisms of its anticancer and anticoagulant abilities are not fully understood, and a more profound comprehension of the interplay between structure and activity is needed [142]. Furthermore, one has to respond to difficulties in controlling Hep’s quality due to its poly component and holistic pharmacologic characteristics [143]. Indeed, Hep’ preparations contaminations have even resulted in patient death [144].

### 3.2. Chondroitin Sulfate and Dermatan Sulfate-Based Nanoparticles as Drug Delivery Systems

CS exhibits high biocompatibility and specific localization, being bound to PGs in ECMs of tissues such as cartilage, blood vessel walls, skin, and tendons [48]. In line with increasing nanotechnology application, optimally designed nano-scaled carriers on the base of CS have been developed, exhibiting unique properties, such as biocompatibility, low toxicity, and active and passive targeting. Their specific properties and discrete modalities make them promising drug delivery vehicles for cancer therapy [145].

Because CS, like all GAGs, is a specific anionic acid polysaccharide, it couples well with cationic poly-saccharides, including chitosan, which as a natural molecule is likewise characterized with good bioactivity [146]. Thus, a CS–chitosan nanoparticle carrier encapsulating black rice anthocyanins exhibited significant apoptosis-inducing effects in colon cancer cells [147], whereas loading these nanoparticles with curcumin induced a cytotoxic effect in the lung cancer model [148].

Moreover, loaded with camptothecin (CPT) polymeric nanoparticles functionalized with CS exerted targeted colon cancer drug delivery with superior anticancer effects compared to non-targeted nanoparticles [149]. This approach utilized CS’s affinity for the CD44 HA receptor overexpressed in various tumors [107].

Notably, CS can lower the adverse side effects of chemotherapeutic drugs as CS-Dox-poly(lactic-co-glycolic acid) (PLGA)conjugated nanoparticles exhibited lower cardiotoxicity and enhanced tumor inhibition compared with free Dox [150]. This development is an important achievement as cardiac toxicity through various mechanisms is a severe drawback of Dox utilization [151,152].

#### Summary

In summary, CS-derived drug-loaded nanomaterial has been shown to have a reasonable encapsulation efficiency, an appropriate hydrodynamic diameter, manageable surface charge, low toxicity, and improved anticancer properties compared to the free drug [149,153,154].

### 3.3. Keratan Sulfate in Anticancer Drug Delivery

KS, another perspective GAG for drug delivery, is localized in the ECMs of different tissues, such as cartilages, cornea, and bone [15]. Besides acting as a constitutive molecule of the ECMs, KS also plays a role as a hydrating and signaling agent in cartilage and cornea tissues. KS chains are structurally bound to a protein core, forming PGs. Unlike other GAGs, KS lacks uronic acid and contains galactose in its disaccharide building blocks. Moreover, the unsulfated Gal residue is essential for binding mediated through non-electrostatic interactions [155], such as hydrophobic and/or van der Waals forces [156].

These data suggest that protein binding strategies may need to be chosen based on the GAG class to be incorporated in the drug delivery vehicle [157,158,159].

#### Summary

Several reviews describe the structures and functions of KS proteoglycans, but their potential role in drug delivery has not yet been determined [157,158,159].

### 3.4. Hyaluronic Acid-Based Nanoparticles for Controlled Drug Release in Cancer

HA is an abundant GAG, deposited to most tissues’ ECM [160]. Its properties, biodegradation, biocompatibility, water-solubility, non-toxicity, and non-immunogenicity and its chemical characteristics, enabling modifications with functional groups, define HA as a suitable molecule carrier to deliver low molecular weight drugs [161]. Furthermore, its specific ligation with cell surface receptors such as CD44 and RHAMM [111] enables HA-based nanoparticles to target diseased cells that express these receptors. Indeed, CD44 and RHAMM receptors are overexpressed by many tumor types [162,163,164]. High production of HA has been determined in many solid tumors, but it is the combination of HA production and Hyal overexpression that facilitate both carcinogenesis and metastasis [165]. In prostate cancer, the increased release of low molecular weight HA (LMWHA) due to Hyal1 overexpression and increased HASs activity results in enhanced autocrine proliferation [166]. The naked mole-rat example, the only mammal resistant to cancer, argues the importance of HA. This rodent produces high amounts of very high molecular weight HA and simultaneously exhibits low Hyals expression, correlated to the minuscule ability to cleave HA [167].

Therefore, the involvement of HA in tumorigenesis processes is of crucial significance. This finding has ignited vibrant research efforts directed at HA metabolism and focusing on the inhibition of HA degradation and on blocking HA-receptors interaction. The HA-degrading enzymes Hyals have been identified as attractive anticancer therapy targets due to their cell surface or extracellular deposition. HA localization enables their inhibition in the ECM [81].

The use of HA-based nanoparticles requires knowledge of HA pharmacokinetics. Thus, it is well established that blood and lymphatic transport system are responsible for HA distribution in the body [168,169]. The utilization of isotopes showed that high molecular weight HA (HMWHA) mainly accumulates in the liver, while LMWHA is secreted in urine [170]. Notably, many studies indicate that the differences in HA-based nanoparticles’ targeting efficiency depend on their molecular weight. For example, HMWHA-coated lipid nanoparticles exhibited a stronger binding affinity to the CD44 receptor of murine melanoma cells in vitro than theLMWHA nanoparticles [162].

**Figure 1:**
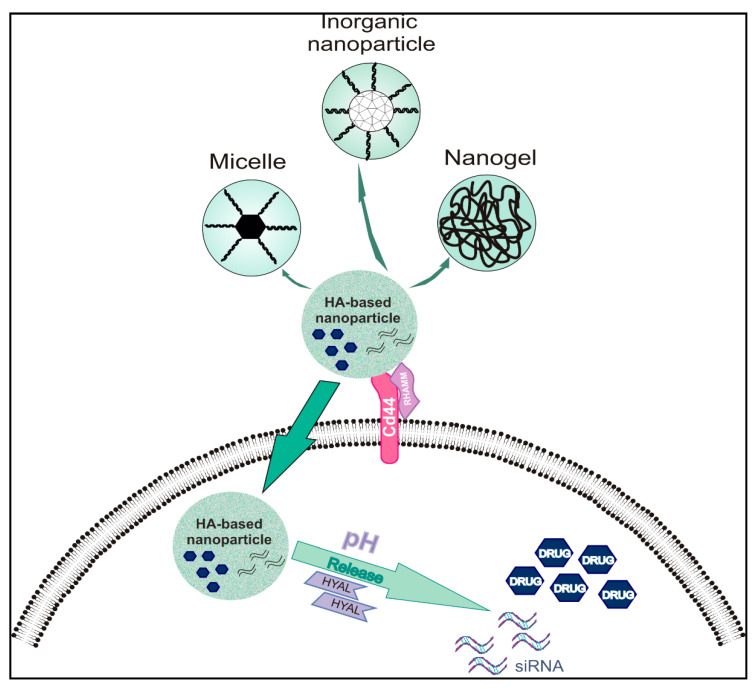
Mechanism of action of HA-based nanoparticles: HA (Hyaluronic acid)-based nanomedicines are used to mediate targeted delivery of therapeutic compounds (DRUGS or siRNA: small interfering RNA) in cancer cells. Nanoparticle targeting is enhanced by HA-specific interaction with CD44 or RHAMM, which are overexpressed in different cancer cell types. These receptors also mediate the internalization of the nanoparticles. After their uptake, each type of nanoparticle is degraded either by enzymatic lysis of HA by hyaluronidase (HYAL) action or by a pH-dependent mechanism.

Different types of HA-based nanoparticles with discrete features have been used as drug carriers (summarized in Figure 1).

#### 3.4.1. Hyaluronic Acid-Based Micelles

HA-based micelles were shown to target CD44 positive breast cancer cells with high-affinity in vitro and in vivo [103]. The hydrophilic HA backbone is modified with hydrophobic groups to form an amphiphilic compound, which can self-assemble into a micelle in an aqueous solution and encapsulate or conjugate drugs After reaching cancer cells, drug release is achieved using various mechanisms, such as through pH dependence or enzyme action [171,172].

HA-conjugated hexadecylamine micelles for the docetaxel delivery to MDA-MB-231 breast cancer cells are examples of a study testing HA-conjugated micelle drug formation [173]. Results showed that HA conjugation of micelles enhanced cellular uptake. Moreover, treating mice bearing xenograft MCF-7 human breast cancer tumors with HA-shelled-paclitaxel prodrug micelles resulted in 100% mouse survival and tumor-specific accumulation of the micelles [174].

In pancreatic cancer, the use of HA-engineered nano-micelles loaded with 3,4-difluorobenzylidene curcumin were tested in CD44-positive MiaPaCa-2 and AsPC-1 pancreatic cancer cell lines [175]. The existence of pancreatic cancer stem cells overexpressing CD44 was identified, contributing to a tumor’s resistance to chemotherapy [175,176]. Recent studies in vivo continued to prove the success of HA:Sucrose nanoparticles in the delivery of anticancer treatments (such asEF2-Kinase inhibitor) to pancreatic cancer cells, leading to significant inhibition of division and tumor formation [177].

Recognized, HA-based micelles’ disadvantages are the limited drug circulation in the blood and the fast uptake by liver endothelial cells [178]. Therefore, the conjugation of HA-micelles with PEG has been tested to improve their blood circulation time. Although PEGylation may affect the micelle interaction with cancer cells HA-receptors [179], the simultaneous use of these two types of micelles showed no significant variances as to their delivery efficiency in vivo [180].

#### 3.4.2. Hyaluronic Acid-Based Nanogels

These types of nanoparticles with physically or chemically crosslinked polymer chains possess pores that can be filled with macromolecules to target cancer cells, as initially demonstrated in vitro [181,182]. HA-based nanogels are used to improve the activity of delivering compounds, enhancing stability, and increasing the biological half-life of HA [178,183]. Indeed, several studies demonstrate the efficiency of HA-based nanogels as drug carriers [184,185]. Moreover, it is possible to link HA with coiled-coil peptide, creating a pH-sensitive nanogel for controled drug release to increase the anti-tumor effect on MCF-7 cells in vitro [186]. Furthermore, it was shown that HA nanogels, fabricated by the methacrylation strategy, are sensitive to enzyme action. The nanogels target cancer cells in a manner dependent on HA receptors expression and are deconstructed by the action of Hyals, releasing their drug load [185]. The introduction of cholesterol to crosslinked nanogels confers hydrophobicity to HA, increasing cell membranes’ permeability to HA-based nanogels [187]. Another way to enhance the hydrophobic features of HA nanogels is to acetylate the HA backbone. Indeed, acetylation’s degree affects Hela cells’ drug loading efficiency and targeting in an in vitro experimental model [188].

#### 3.4.3. Inorganic Hyaluronic Acid-Based Nanoparticles

Another category of HA-based nanoparticles is the metal-organic framework NPs conjugated with HA. These porous materials carry many metal-binding sites that can be used for specific functionalization [106,179]. Their advantages are connected to their high drug loading efficiency due to increased binding surface area [189]. This type of NP is sensitive to different pH conditions, allowing fine-tuning of their release, as demonstrated in a prostate cancer cell model [190].

Combining metal with HA allows the exploitation of specific characteristics of both materials. Thus, AuNPs improve radiotherapy due to gold’s ability to adsorb X-rays, demonstrated in animal models [191]. On the other hand, HA allows the restructuring of AuNPs surface, enhancing the ability of the hybrid NPs to conjugate with drugs [192]. Moreover, the combined NPs exhibit superior stability and high-affinity targeting of CD44 positive liver cancer cells in vitro, as shown by Kumar et al. [193]. Furthermore, Dox-HA-super-paramagnetic iron oxide nanoparticles (Dox-HA-SPION) were suggested to enhance the drug efficacy and to minimize off-target effects in MDA-MB-231 human triple-negative breast cancer cells (TNBC) [194]. Liu et al. designed tumor-targeting HA-titanium dioxide (HA-TiO2) nanoparticles loaded with cisplatin (CDDP) with significant anticancer activity in the A2780 ovarian cancer cells [195]. Silica NPs, also classified as inorganic nanomedicines, have many advantages, such as controllable shape and size, low toxicity, and good biocompatibility [196]. Modified with HA silica NPs exhibit increased delivery to HA-receptor expressing cancer cells, as demonstrated in vitro and in vivo [197].

#### 3.4.4. Clinical Trials Implementing Hyaluronic Acid-Based Nanoparticles

Primary studies in cell cultures and animal trials have shown promising results of HA-based nanoparticle efficiency in anticancer therapy. Some of these compounds have already been tested in Phase 2 or Phase 3 clinical trials with positive outcomes regarding efficiency and safety. A phase 2 trial tested HA-irinotecan plus cetuximab in 45 patients with KRAS wild-type metastatic colon cancer to examine the compound’s safety and efficacy. However, the results of the study have not yet been announced [198]. Another phase 2 study involving 39 patients with extensive-stage SCLC indicates that HA-irinotecan treatment provides survival benefits for patients bearing CD44 positive tumors [199].

Furthermore, phase 1 and 2 clinical trials utilizing HA-cisplatin nanoconjugates (HA-Pt) in dogs with naturally occurring anal sac carcinoma, oral squamous cell carcinoma, oral melanoma, nasal carcinoma, or digital squamous cell carcinoma have been conducted. The obtained results demonstrated the beneficial effects of HA-Pt drug formulations for the treatment of canine squamous cell carcinomas. Moreover, nephrotoxicity, a serious side-effect of Pt therapy, was not evident in any canine subject. Notably, canine oral SCC’s similarity to human HNSCC regarding progression and drug response gives essential information for developing human treatments [200]. Examples of HA-based nanoparticle types tested in different cancer models are shown in Table 2.

## 4. Targeting GAGs in Cancer—New Prospective

### 4.1. Targeting Heparan Sulfate/Heparin

HS, expressed by all mammalian cells in homeostasis [31], has been determined to be the most complex GAG [19]. This highly variable GAG is critical in cellular signaling and extensively remodeled during cancer progression. In its natural state, Hep is a heterogeneous mixture composed of polysaccharide chains that exhibit varying lengths and different sulfation patterns. Hep, compared to HS, is more homogeneous and its main function is storage. HS and Hep chains can establish specific interactions with various protein mediators regulating critical cellular signaling [18]. The affinity of HS/Hep chains to proteins such as growth factors seems to be significantly affected by their sulfation status and resulting electrostatic interactions [157,158,159]. Moreover, analysis by the polyelectrolyte theory demonstrated that the binding of FGF-2 to Hep is primarily accomplished through the more specific nonionic interactions, such as van der Waals packing and hydrogen bonding [201]. Therefore, inherent properties of the GAG chains need to be taken into account when designing novel drug carriers [157,158,159].

To date, more than 400 HS-binding proteins have been identified, including cytokines, growth factors, chemokines, ECM proteins, as well as enzymes and enzyme inhibitors [18]. Thus, the targeting of HS protein interactions is an essential developing therapy approach.

The strategies that have been examined for cancer-oriented therapy are based on (i) the utilization of GAG mimetics as competitive agents to block HS–protein interactions (ii) the utilization of enzymatic methods to cleave or modify HS to inhibit HS–protein interactions.

The utilization of unfractionated Hep and LMWHs is standard clinical practice for the protection against venous thromboembolism in cancer patients [202]. This clinical practice’s implementation has also demonstrated a beneficial effect of Hep on cancer patient survival discrete from its anticoagulant properties [203]. Indeed, Hep has now been recognized as a multifunctional drug [50]. Hep mimetics are commonly described as synthetic or semi-synthetic compounds that are anionic, usually highly sulfated, and structurally defined as distinct GAG analogs [204].

Research efforts focused on the synthesis of Hep derivatives with attenuated polypharmacy traits and anticoagulant activity, exhibiting enhanced potency and specificity while downregulating unwanted side effects, e.g., anticoagulation [204]. This approach has been facilitated by significant development in carbohydrate synthesis, including one-pot multi-step procedures and coupling reactions, enabling the synthesis of complex oligosaccharides [205].

A recently synthesized, multitargeting Hep-based mimetic, necuparanib, was shown to attenuate pancreatic cancer tumor cell growth and invasion in a three-dimensional (3D) culture model. In contrast, in vivo, it facilitated survival and attenuated the metastatic ability of pancreatic cancer cells. Furthermore, the proteomic analysis demonstrated that necuparinib, among others, targeted ECM-originating mediators, well established to affect cancer cell growth and metastasis. Specifically, necuparanib attenuated the expression of metalloproteinase 1 (MMP1) and facilitated the expression of tissue inhibitor of metalloproteinase 3 (TIMP3) in the 3D pancreatic cancer model [206]. Moreover, the levels of TIMP3 in the plasma of patients with metastatic pancreatic cancer who were participating in a phase I/II study treatment with necuparanib plus standard therapy were found to be substantially enhanced [206].

A crucial therapeutic target is cancer-associated angiogenesis. Both fibroblast growth factors (FGFs) and vascular endothelial growth factor (VEGF) can form ternary complexes with HS and their respective cell-membrane receptors, initiating signaling cascades that facilitate angiogenesis [207]. These growth factors are characterized as important cancer therapy targets with Hep mimetics’ possible implementation [208,209]. The d-mannose-based sulfated oligosaccharide mixture, PI-88 (Muparfostat) is one such inhibitor. It is developed from the oligosaccharide phosphate fraction obtained from the extracellular phosphomannan, initially derived from the yeast *Pichia (Hansenula) holstii* (NRRL Y-2448) and subsequently extensively sulfated [210,211].

Modified LMWH functionalized by polystyrene (NAC-HCPS) exhibited increased affinity to HS binding growth factors and attenuated anticoagulant properties, decreased endothelial cell growth, and formation of endothelial tubes [212]. Moreover, SST0001 Hep derivatives, characterized by 100% N-acetylated, 25% glycol split Hep SST0001 (100NA-ROH, roneparstat), efficiently reduced FGF2-mediated proliferation of endothelial and lymphoid cells and displayed a limited capacity to release FGF from the ECM. This effect is associated with the N-acetylation of GlcN.SST0001 and was also reported to counteract human sarcoma cell invasion induced by exogenous FGF2 [213]. Interestingly, Hep is actively uptaken by melanoma cells and affects their migration and adhesion [214].

The disadvantages of using Hep derivatives, discussed above, are mostly correlated to the intrinsic Hep anticoagulant properties to initiate severe hemorrhagic effects.

### 4.2. Enzymatic Modulation of HS–Protein Interactions

Heparanase, the only mammalian enzyme responsible for HS/Hep cleavage, is a strict endo-β-glucuronidase, favoring the fission of a GlcA linked to 6O-sulfated GlcN, which can either be N-sulfated or N-acetylated [56]. However, advances have implicated the potential controlling role of the surrounding saccharide sequences and their sulfation pattern in regulating the extent of substrate degradation [56].This plasticity of substrate specificity enhances the execution of various heparanases’ roles [215]. The cleavage of HS chains bound into PGs releases latent growth factors, including FGF2, hepatocyte growth factor (HGF), keratinocyte growth factor (FGF4), and TGF-β, which are sequestered to the matrix and cell surface, but also inherently modulates the protein-GAG interactions and downstream signaling [216]. Indeed, trimming of HS can enhance the binding of growth factors to their respective receptors, as in the case of FGF-2 where the creation of tertiary FGF2-FGFR-HS complex is increased by moderate heparanase activity [217]. Moreover, heparanase was found to reside and accumulate in lysosomes suggesting that it also exhibits intracellular functions [218].

Heparanase strongly affects protein–HS interactions, whereas tumor-associated activated fibroblasts, endothelial cells, and immune cells exhibit increased heparanase activity [219]. The overexpression of heparanase results in vivo in increased tumor metastasis, whereas downregulating heparanase markedly decreases cancer cells’ ability to metastasize [220].

Heparanase expression was shown to be upregulated in all cancer types, including sarcomas, carcinomas, and hematological neoplasms [221]. Notably, heparanase activity has been correlated to various human cancers’ metastatic potential. Thus, the examination of the Cancer Genome Atlas (TCGA) data on heparanase expression in breast cancer clinical samples showed its upregulation in the majority of specimens. Furthermore, increased heparanase expression was correlated with poor patient survival [222]. Similar results have been obtained for other cancer types, including multiple myeloma [223] and bladder cancer [224]. Moreover, heparanase has been shown to affect cancer angiogenesis [225], invasion, and autophagy [226] and partly through syndecan-1-dependent mechanisms to modulate inflammation-associated tumorigenesis [227].

Heparanase can affect the response to chemotherapy. Thus, anti-myeloma chemotherapeutic agents, including bortezomib (proteasome inhibitor) or melphalan (alkylating agent), were shown to increase the expression and secretion of heparanase in an in vitro myeloma model. The subsequent uptake of soluble heparanase by tumor cells initiated ERK and Akt signaling pathways, stimulated the expression of vascular endothelial growth factor (VEGF), HGF, and MMP-9, and was correlated with an aggressive tumor phenotype [228].

An essential mechanism of heparanase action is promoting exosome secretion, which affects both tumor and host cells’ biological behavior and finally drives tumor progression [229]. In a myeloma model, it was shown that chemotherapeutic drugs increase exosome secretion. Notably, chemoexosomes have an increased heparanase load, enhancing cell HS’s cleaving activity and initiating ERK signaling and syndecan-1 shedding. These authors suggest that anti-myeloma therapy stimulates the secretion of high heparanase content exosomes, facilitates ECM remodeling, changes tumor and stroma cell behavior, and contributes to chemoresistance [230].

Several therapeutic approaches have been tested to develop efficient inhibitors of heparanase activity. Non-anticoagulant heparin derivatives such as SST0001 or roneparstat significantly downregulated heparanase-dependent cleavage of syndecan-1 HS chains, attenuated HGF, VEGF, and MMP-9 expression resulting in decreased tumor growth and angiogenesisinvivo [231,232]. Preclinical evidence resulted in the first human study (NCT01764880) assessing the safety and tolerability of roneparstat in patients with relapsed or refractory multiple myeloma (MM). Patients treated with Roneparstat exhibited acceptable tolerance at clinically significant doses [233].

PI-88 is an inhibitor of heparanase, in addition to its antagonist of angiogenic growth factors function [234]. Even though it exerted adjuvant properties in hepatocellular carcinoma and melanoma patients [235,236], PI88 has been correlated with bleeding events, and thus, did not progress to clinical practice [237].

A series of PI-88 analogs have been synthesized, exhibiting superior performance. The improved analogs comprise single, characterized oligosaccharides with discrete functionalizations and exhibit more efficient antagonism of angiogenic growth factors and respective receptors binding with HS. These properties are translated into potent inhibition of growth factor-dependent endothelial cell growth and strong downregulation of the endothelial tube formation [234]. PG545 is the outstanding member of the PI88 analogs series exhibiting significant anti-angiogenic, anti-proliferation, and antimetastatic effects through potent heparanase inhibitory and angiogenic growth factor antagonist effects [238]. Moreover, PG545 was shown to exert anti-tumor effects discrete from heparanase inhibition as it induces lymphoma cell apoptosis in a non-heparanase-dependent manner [239]. PG545 (pixatimod) is currently being tested in clinical trials [238]. However, despite promising breakthroughs, the development of heparanase inhibitors with beneficial clinical performance and acceptable adverse effects is still elusive. Therapeutics targeting HS are summarized in Table 3.

However, some studies targeting heparanase demonstrated contradictory results. In some model systems, inactive heparanase facilitated adhesion and migration of endothelial cells and induced factors that promote angiogenesis, such as vascular endothelial growth factor [240]. The enzyme has a C-terminus domain involved in the molecule’s signaling capacity. The human heparanase variant (T5) lacking enzymatic activity has protumorigenic properties, indicating the enzyme’s complex role in cancer pathogenesis [240].

## 5. GAGs and Immunological Aspects of Cancer Therapy

The involvement of glycobiology in cancer and the anti-tumoral immune response can be analyzed at several levels. GAGs are involved in the immune response; they can constitute new biomarkers and offer possibilities to develop new immune-therapy targets.

The interconnection of the immune system and various aspects of tumorigenesis are described in all types of cancers [241,242]. An array of immune cells, mainly from the myeloid lineage, macrophages, and dendritic cells, modulate tumor neoangiogenesis. HA is an essential component of the TME, and its abnormal deposition has been assessed in different tumor types. As HA is one of the modulators of tumor angiogenesis, it can influence various immune cells’ physiopathology within the TME. HA-induced effects depend on both its polymer size and its complexes with other molecules. Under healthy conditions, HASs and Hyals are a tightly regulated molecular network that keeps HA ECM levels within physiological limits. When pathological conditions appear, and HA homeostasis is perturbed, the enzymes that regulate its characteristics aid the pro-tumoral processes in TME and induce resistance to therapy [243].

Inflammation and tumorigenesis are intertwined processes [244], and inflammatory mechanisms are involved in both the tumors’ initiation and progression. In the continuous communication with the ECM, GAGs regulate the cell/matrix interface and the immune-related mechanisms [2].

### 5.1. GAGs Roles in Tumor Immunology

CD44-HA constitutes a molecular tandem that can affect tumor immunology by utilizing complex mechanisms [6]. Macrophages have a different expression of CD44 related to their functions, and their capacity to bind HA is variable. CD44 has the highest expression in M1 polarized macrophages, followed by M0 type of macrophages, whereas M2 type expression is similar to the latter. The higher CD44 expression in M1 induces increased binding of HA. On the other hand, the lower M2 expression favors a better internalization of HA. Therefore, the molecular mechanisms in the CD44-HA tandem exhibit subtleties to predict targeting behavior [245].

The presence of tumor-associated macrophages (TAMs) is correlated with the poor outcome in tumor-bearing organisms because these cells sustain the immune-suppression and enhance pro-tumoral mechanisms, and, last but not least, inhibit the actions of anti-tumoral drugs. Therefore, TAMs are preferred targets in tumor therapies [246]. Several years ago, a nanoparticle was designed comprising poly(lactic acid-co-glycolic acid-grafted HA (HA-g-PLGA) that carried a cytostatic, an active metabolite of irinotecan. At the acidic pH of the TME, HA is exposed, and by linking to the CD44 expressed on tumor cells and TAMs, it delivers the cytostatic intracellularly. Tumor cells continue to recruit TAMs that encounter carriers with the cytostatics; hence, the anti-proliferative effect is propagated [247].

In a recent study, novel carrier molecules were tested. Within the tested compounds, oligomeric HA (oHA) targeted CD44 receptors on TAMs for the delivery of curcumin (Cur) and baicalin (Bai) to overcome tumor resistance. The carrier had good cellular penetration and cytotoxicity upon tumor cells. In in vivo animal modelsof A549 tumor-bearing nude mice, the significant anti-tumoral effect was re-confirmed [248]. HA-based nanoparticles were tested as drug carriers in epithelial ovarian cancers to target TAMs specifically. Thus, HA nanoparticles that encapsulate miR-125b (HA-PEI-miR-125b) targeted TAMs in an experimental mouse model of syngeneic ID8-VEGF ovarian cancer and induced these cells to an immune-activating phenotype [249]. In the 4T1 breast cancer animal model, mesoporous Prussian blue (MPB) nanoparticles and LMWHA (LMWHA-MPB) were tested. This approach demonstrated that LMWHA-MPB penetrates M2 macrophages (pro-tumoral macrophages), which are subsequently diverted toward the M1 phenotype exhibiting anti-tumoral action. Therefore, LMWHA-MPB can induce TAMs pro-tumoral potential and can likewise be used in situ for microenvironmental tumoral regulation [250].

LMWHA per se was demonstrated to have an anti-tumoral effect in colorectal carcinoma. The immune response involves activated dendritic cells (DC). Authors have shown that preconditioning DC from tumors with LMWHA increased their ability to migrate in vitro and enhanced DC in vivo recruitment to regional lymph nodes. In a mouse animal model, tumor lysate-pulsed DC (DC/LMWHA) was administered, and a potent anti-tumor response was obtained. Splenocytes from animals treated with DC/LMWHA displayed higher proliferative capacity, enhanced IFN-γ production, and lower immunosuppressive cytokine levels. Therefore, LMWHA can be considered a new adjuvant candidate for DC-based anticancer vaccines [251].

Using HA’s ability in reprogramming pro-tumoral M2 type TAMs to anti-tumor M1 macrophages, other nanoparticles with MnO2 were used to decrease tumor hypoxia chemoresistance in the breast cancer experimental model. Increased tumor oxygenation was obtained in conjunction with hypoxia-inducible factor-1 α (HIF-1α) and VEGF downregulation. When these nanoparticles were combined with classical cytostatic Dox, tumor growth/proliferation was inhibited [252].

As cancer immunotherapy has recently gained unprecedented momentum, HA’s involvement as a drug carrier was tested. TC-1, a polymeric conjugate formed by HA and ovalbumin (OVA) as a foreign antigen, was tested using mouse lung tumor cells. This model showed that OVA257-264 peptide is presented complexed with MHC class I on the cells’ surface. With this approach, the foreign antigen could induce an anti-tumor effect by enhancing the immune cells’ attack. The mouse model’s systemic administration showed that the conjugate is accumulated into tumor tissue and facilitates the cytotoxic T lymphocytes’ (CTLs) attack of the tumor cells, thus inhibiting tumor proliferation [253].

OVA-loaded micelle consisting of PEGylated HA was tested for increasing the OVA uptake. The HA-coated micelle targeted CD44 on tumor cells and increased OVA cellular uptake more than 10 times. Loading tumor cells with a foreign antigen, such as OVA, would increase their recognition by CTLs, and thus, enhance destruction. In animal models, tumor growth was significantly inhibited, and the authors point out that in the case of cutaneous melanoma, this can be another approach to enhance immune-therapy [254]. The same principle was implemented in TC-1 mouse cells and lung cancer epithelial cells, using MMP9-responsive conjugates consisting of PEGylated HA and OVA. The complex was taken up through CD44-expressing cancer cells via receptor-mediated endocytosis. In an in vivo animal model, the tumor growth was significantly inhibited, antigen presentation on the tumor cells enhanced, and T cytotoxic anti-tumoral action increased [255]. A complex using HA and OVA on gold nanoparticles (AuNPs) was used to increase antigen uptake, by DC, via receptor-mediated endocytosis. The complex HA-OVA-AuNPs has enhanced near-infrared (NIR) absorption and thermal energy translation, so after engulfment, the cytosolic antigen will be delivered through the photothermally targeted process. Proteasome activity is increased, and the MHC I antigen presentation is enhanced; thus, the CD8+ cytotoxic T-cell response is triggered. This protocol can be fruitfully expanded in the cancer vaccine development area [256].

**Figure 2:**
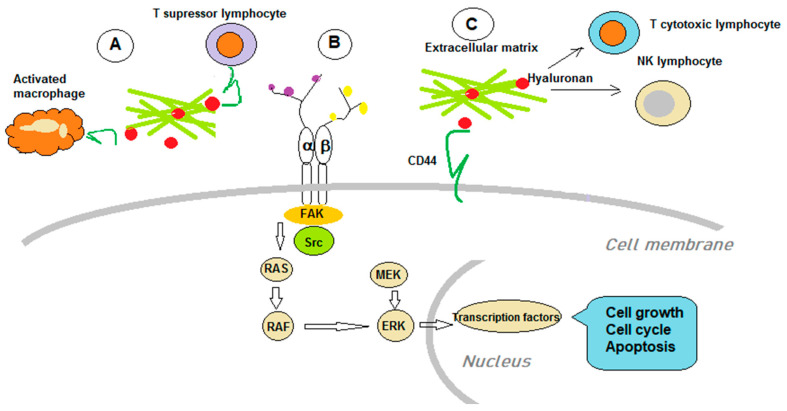
The main mechanisms through which GAGs hinder immune anti-tumoral action. A. HA binds t oCD44-expressing T suppresor cells and to the pool of tumor-associated macrophages contributing to the immuno-suppressive milieu in TME; B. Specific enzymes (e.g., β1,4-N-acetylgalactosaminyltransferase 3 and β1,4-galactosyltransferase 3) induce modification of β1 integrin expressed by tumor cells, triggering intracellular signaling that favor pro-tumorigenic effects in cell growth, cell cycle, and apoptosis. C. HA in the TME binds toCD44 expressed by tumor cells to physically block NK and T cytotoxic lymphocytes’ access to tumor cells.

As some of the tumor cells and primary lymphocytes have low HS expression, other carriers need to be utilized. Thus, proteins complexed with nanosize cholesteryl group-bearing pullulans (cCHP) can be efficiently delivered to myeloma cells and to primary CD4+ T cells by macropinocytosis. When using these new types of nanoparticles to deliver the anti-apoptotic protein Bcl-xL, T cells’ functional regulation is achieved. These nanoparticles can bypass the lack of HS expression and deliver anticancer effectors and modulators of immune regulation [257].Figure 2 outlines the main mechanisms GAGs utilize to hinder immune anti-tumoral action.

### 5.2. GAGs as Immunotherapy Targets

TME is complex and consists of immune cells (mainly lymphocytes and myeloid cells), non-immune cells (mainly endothelial cells and fibroblasts), and a complex array of structures, such as ECM, and various molecules that are either secreted or append to the cell membrane [258]. TME sustains molecules that hinder the potential effector function of NK lymphocytes. Transforming growth factor (TGF)-β and members of its superfamily downregulate NK cell cytotoxicity functions, cytokine secretion, metabolism, and proliferation. Likewise, galectins, a family of carbohydrate-binding proteins produced by different sources within the TME, downregulate NK cell functions. Various ECM components and associated enzymes (e.g., MMPs) can hinder NK cells’ activation and become future therapy targets [259]. Pancreatic cancer, TME, contains various possible therapy targets, such as HA, focal adhesion kinase (FAK), connective tissue growth factor (CTGF), CD40, and chemokine (C-X-C motif) receptor 4 (CXCR-4), which could be utilized in future clinical applications [260].

Immune checkpoint inhibitor immunotherapies that had achieved broad clinical applicability in recent years [261] face the gaining of resistance. It is supposed that the HA accumulation influences tumor cells’ sensitivity to chemotherapy and immunotherapy. A semiquantitative grouping of non-small lung cancer tissue demonstrated that HA deposition predicts the tumor response to pegylated hyaluronidase (PEGPH20) in animal models [262]. Thus, HA degradation facilitates tumor cells’ exposure to drugs. Notably, utilization of PEGPH20, in a phase I clinical study demonstrated safety and good tolerability [263]. A phase I clinical trial, combining PEGPH20 with an immunotherapeutic agent, pembrolizumab, is currently ongoing in a cohort of metastatic gastric adenocarcinoma and non-small cell lung carcinoma patients [264], The reasoning behind this approach is the combination of facilitating drug access to tumor cells with the hypothesis that HA may modulate regulatory T cells and antitumor immune responses [265]. Clift et al. have shown that upon degrading HA, the anti-programmed death-ligand 1 (PD-L1) antibody accumulates more intensely in breast cancer tissues in vivo. An increased accumulation of T and NK cells was noticed upon HA degradation. The authors point out that decreasing HA in TME would enhance anti-tumoral immune cell infiltration and increase checkpoint inhibitor therapy efficacy [266].

Heparanase has also been linked to tumor immunology. It was shown that heparanase is implicated in chronic inflammatory bowel conditions and, consequently, in colon carcinoma initiation [222,223,224]. There is a clear correlation between intestinal heparanase and immune cells, mainly macrophages, which sustain the chronic inflammation and create a pro-tumoral microenvironment. Therapies that can re-equilibrate this enzyme’s function and re-establish the physiological crosstalk between immune and epithelial cells would hinder colon cancer development [267]. Leukocyte-derived heparanase is versatile; therefore, subtle changes in the TME can direct the enzyme to either pro-or anti-tumoral action. Thus, in immune cancer therapy, heparanase could be a vital therapy target by either exploiting or inhibiting its activity [268].

Along these lines, heparanase inhibitors were tested in various hematological cancer models. Weissmann et al. showed in 2019 that PG545, a heparanase inhibitor, had a strong effect on human lymphoma. The inhibitor induces tumor cell apoptosis, ER stress response, and increased autophagy. PG545 did not affect naïve splenocytes but induced apoptosis even in lymphoma cells deployed of heparanase activity [239]. Another approach was utilizing heparanase-neutralizing monoclonal antibodies that strongly attenuate lymphoma cell tumor load in mouse bones due to tumor cell growth inhibition and reduced angiogenesis [269].

In chronic lymphocytic leukemia (CLL), stromal cells secrete and present CXCL12, a CXC chemokine ligand, through cellsurface-bound GAGs. By using this mechanism, CLL cells are protected from cytotoxic drugs and sustain the residual disease. The GAG mimetic, NOX-A12, binds and neutralizes CXCL12 and was tested to affect tumor cell migration. NOX-A12 inhibited CLL cell chemotaxis generated through CXCL12. Thus, NOX-A12 competes with GAGs (e.g., Hep) for CXCL12 binding and sensitizes CLL cells toward chemotherapeutic drugs [270]. An outline of the main immune-therapy targets is summarized in Table 4.

## 6. GAGs as Potential Cancer Therapy Response Biomarkers

The physical barrier represented by HA in the TME restricts immune therapy efficacy by hindering antibody and immune cell access. It was shown in 50% of HER2(3+) primary breast tumors and almost 50% of EGFR(+) head and neck squamous cell carcinomas that the tumor tissue characterized by high HA expression is associated with immune therapy resistance. The matrix containing high HA deposition hinders NK immune cell access to tumor cells. The depletion of HA by PEGPH20 (pegylated recombinant human PH20 hyaluronidase) propagates NK cells’ access to these tumors. In vitro, the same mechanisms enhanced trastuzumab- or cetuximab-dependent antibody-dependent cellular toxicity (ADCC), while the in vivo experiments also demonstrated treatment efficacy. Considering that the tumor HA deposition can be used as a marker for immune therapy resistance, other clinical management protocols can be developed [271].

In colorectal cancer, it was established that glycosylation alters over 80% of human proteins and that aberrant glycosylation is involved in cancer development and progression. Glycan changes (e.g.,carbohydrate antigen CA 19-9 or carcinoembryonic antigen) are already established biomarkers in this cancer. Recent reports have shown that altered glycosylations can be involved in drug resistance mechanisms and indicate new predictive biomarkers [272].

GAGs are utilized as biomarkers in other disease types, including mucopolysaccharidoses (MPSs) [273]. The MPSs present approximately 30% of lysosomal storage diseases and are induced by inefficient GAG breakdown due to active enzyme deficiencies [274]. Without treatment options, patients exhibiting severe MPS forms die within the first two decades of life [273].

## 7. Conclusions

GAGs are versatile molecules that play multifaceted roles in the human body. They are involved in all biological functions and are acrucial mediator of homeostasis. Alterations in both the expression and GAG fine chemical structure are evident during cancer development and progression. Research efforts directed at the role of GAGs in cancerogenesis are rapidly increasing, and some of the findings have made their way into clinical practice.

The field has been facilitated by essential developments in available technologies, including imaging technologies, mass spectrometry, microarrays, and bioinformatics tools [275,276,277]. Therefore, we can now deepen our studies of the glycome, leading to an improved understanding of the glycobiology field. Indeed, the recent advancements in the GAG structure/function relationship have allowed a better appreciation of the GAGs role in tumorigenesis and the utilization of this knowledge for cancer detection, prognosis, and therapy implementation. GAGs are now being employed as biomarkers for disease progression and tumor aggressiveness [278].They are involved in the tumor immune response, can be used by themselves or in the form of hybrid PGs therapeutic targets, and offer targeted drug delivery [1,279]. As drug carriers, GAGs are characterized by high specificity, multi-functionality, and good biocompatibility, the key to the success of new therapies in oncology [279]. Considering that GAGs are critical molecules of the complex cellular and molecular TME network, their multi-factorial utilization could enable personalized therapy implementation. However, some obstacles still need to be overcome as the heterogeneity of native GAG preparations has introduced the need for producing synthetic or semi-synthetic GAG mimetics with improved pharmacokinetic properties, higher selectivity, and attenuated or even abolished adverse side-effects. Future research efforts will enhance GAG implementations in the clinic and hopefully improve therapeutic strategies for some cancer types.

## Figures and Tables

**Table 2:** Types of HA-based nanoparticles tested in different cancer models.

HA-Based NP Types	Composition	Drug/Conjugate	Human Cancer Type	Reference
Micelles	HA-b-dendritic oligoglycerol	paclitaxel	breast	[174]
	HA-copoly(styrene maleic acid)	3,4-difluorobenzylidene curcumin	pancreatic	[175]
Nanogels	Coiled-coil-peptide-cross-linked-HA	GY(EIAALEK)3GC (E3) and GY(KIAALKE)3GC (K3)	breast	[186]
	Acetylated HA with low molecular weight 1,2,3-with degrees of acetylation 0.8, 2.1, 2.6 acetyl groups per unit (2 glucose rings)	Doxorubicin	cervical	[188]
Inorganic	HA super-paramagnetic iron oxide	Doxorubicin	breast	[194]
	HA-titanium dioxide	Cisplatin	ovarian	[195]

**Table 3:** Therapeutics targeting HS.

Therapy Target	Drug	Cancer Type	Stage	Reference
Antagonists of angiogenic growth factors	necuparanib	Pancreatic cancer	3D model, animal tumor model, Phase I/II clinical trial in combination with standard therapy	[206]
	PI-88 (muparfosfat)	General tumor angiogenesis	In vitro, animal models	[210,211]
	NAC-HCPS	Lung tumor	Animal model	[212]
	Hep SST0001 (roneparstat)	Sarcoma	Animal models Section 2	[213]
Heparanase Inhibitors Section 3	SST0001 (roneparstat)	Multiple myeloma Section 4	Animal model, Clinical trial Section 5	[232,233]
	PI-88 (muparfosfat)	Hepatocellular Carcinoma. melanoma	Clinical trial	[235,236]
	PI-88 analogs (PC545-pixatimod)	Human lymphoma	Animal model, Clinical trial	[237,238]

**Table 4:** Developing GAG-associated immune-therapies.

Target	Therapy	Cancer Type	Stage	Reference
Hyaluronan	PEGylated recombinant hyaluronidase Section 6	Solid tumors	phase I study	[263]
		Non-small lung cancer	Animal model	[262]
		Refractory locally advanced or metastatic gastric adenocarcinoma and Non-small cell lung carcinoma	A phase 1b trial of PEGPH20 with pembrolizumab (NCT02563548)	[264]
Heparanase	Heparanase inhibitors	Colon carcinoma	Animal model	[267,271]
		Human lymphoma	In vitro cellular model	[239]
	Heparanase neutralizing antibody	Human follicular and diffused non-Hodgkin’s B-lymphomas	Animal model	[269]
